# Dietary Pseudopurpurin Effects on Bone Mineral Density and Bone Geometry Architecture in Rats

**DOI:** 10.3390/ijms13033431

**Published:** 2012-03-13

**Authors:** Chen-Chen Wu, Xiao-Bing Li, Tie-Suo Han, Peng Li, Guo-Wen Liu, Wei-Zhong Wang, Zhe Wang

**Affiliations:** 1 College of Animal Science and Veterinary Medicine, Jilin University, Changchun 130062, China; E-Mails: dandan2niuniu@126.com (C.-C.W.); lixiaobingvip@163.com (X.-B.L.); hts-820228@163.com (T.-S.H.); lipeng790625@163.com (P.L.); 2 College of Animal Food and Science Technology, Yunnan Agriculture University, Kunming 650201, China; E-Mail: wucen95888@163.com

**Keywords:** pseudopurpurin, bone mineral density, bone mineral elements, bone geometry architecture

## Abstract

The objective of our study was to evaluate whether feeding pseudopurpurin affects bone mineral density and bone geometry architecture in rats. Pseudopurpurin was extracted, analyzed and purified using an HLPC-ESI-MS. Rats were given 0% and 0.5% pseudopurpurin powder in their diet. Femurs of rats were examined at 0.5, 1 and 2 months after pseudopurpurin feeding. Compared with rats in the group 0%, the bone mineral density, and the calcium, magnesium, zinc and manganese concentrations in the rats femur in the group 0.5% increased significantly at 1 month and 2 months after pseudopurpurin feeding. Analytical results of micro-computed tomography showed that the group 0.5% displayed an increase in the trabecular volume fraction, trabecular thickness and trabecular number of the distal femur at 1 and 2 months after pseudopurpurin feeding, and the mean thickness, inner perimeter, outer perimeter, and area of the femur diaphysis were significantly increased at 2 months after pseudopurpurin feeding compared with the group 0%. In parallel, the trabecular separation and structure model index of the distal femur were decreased, compared with the group 0% at 1 and 2 months after pseudopurpurin feeding. In the 0.5% and 0% groups, there was no damage to kidney and liver by histopathology analysis. The long-term feeding of pseudopurpurin is safe for rats. The feeding of 0.5% pseudopurpurin which has specific chemical affinities for calcium for bone improvement and level of bone mineral density, enhances the geometry architecture compared with the 0% group.

## 1. Introduction

Madder dyes impart a rich scarlet color to cloth and have been used since the third millennium BC. The specific vital staining of developing bone by feeding the dyestuff madder has been common knowledge for hundreds of years [1–7]. Anthraquinones, present in madder roots (roots and rootstocks of *Rubia tinctorum* L.), have proved to exert different biological activities [8], such as anti-oxidant, anti-microbial, anti-fungal, cytotoxic, larvicidal and anti-viral activities [9]. Under normal conditions of madder feeding, the coloration is due to the staining of bone salts by the active components of madder: alizarin, purpurin and pseudopurpurin—pseudopurpurin being the most important—with these dyestuffs become effective when combined with calcium [10]. However, reports on the influence of alizarin on bone growth are confusing. From the available literature, it is known that alizarin does not cause retardation of growth of dentine and bone in the rat [11], and there is a temporary retardation or even a cessation in growth of the bones of the rabbit [12]. Pseudopurpurin resembles alizarin because it forms a colored metal salt that is highly insoluble in water [13], yet, as a material for vital staining of the bones in animals, pseudopurpurin has rarely been used. Richter found that madder contained considerable quantities of pseudopurpurin, and considered that it was responsible for the vital staining of the bones of animals fed on madder, and was completely non-toxic to animals [14]. However, up to now, no experimental study has been carried out on pseudopurpurin’s use in bone mineralization. This therefore needs to be subjected to an in-depth study. With this in mind, we extracted pseudopurpurin from madder, then evaluated whether it affected bone mineral elements and bone geometry architecture in rats during the bone growth process, with the aim of providing further new insights into the effect of pseudopurpurin on bone mineralization in humans and mammals.

## 2. Results and Discussion

### 2.1. ESI-MS Analyses

**Figure 1:**
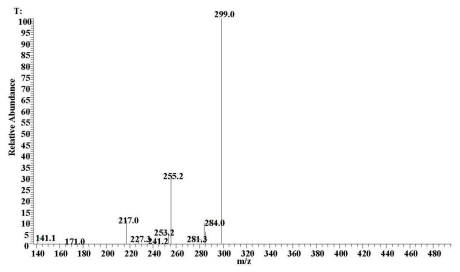
Negative-ion mass spectra of pseudopurpurin obtained by LC-ESI-MS analysis of an extract of madder powder.

Because samples of these carboxylated anthraquinones were not commercially available, confirmation of their identity was achieved by analysis of madder root powder with extraction using the described method. Identification of pseudopurpurin in this extract confirmed that the extraction conditions were non-degradative and suitable for this kind of fragile anthraquinone colorant. The colorant was also observed by monitoring the deprotonated molecule [M–H]^−^, *m/z* 299.8 and the [M–H–CO_2_]^−^ fragment ion at *m/z* 255.2 (Figure 1). The high precision of the mass measurements of these ions, together in comparison with the MS and UV-visible spectra described by Derksen *et al.* [4] and the UV-visible spectra described by Schweppe [15], allowed us to propose the structure of pseudopurpurin.

### 2.2. Body Weight and Femur Length in Rats

There were no significant differences in body weight and femur length in the 0% and 0.5% groups from 0.5 to 2 months after pseudopurpurin feeding (*P >* 0.05), however, the body weight and femur length in the 0.5% group rats were higher than in the 0% groups rats (Table 1).

### 2.3. Bone Mineral Composition and Bone Mineral Density

**Figure 2:**
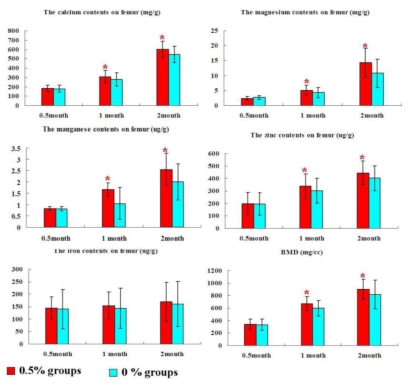
The level of bone mineral composition and bone mineral density in the rat femur in the groups 0% and 0.5% at 0.5, 1 and 2 months after pseudopurpurin feeding. Data are expressed as the means ± SD; * *P <* 0.05 *versus* the group 0%.

At 0.5 month after pseudopurpurin feeding, calcium (Ca), magnesium (Mg), zinc (Zn), manganese (Mn), and iron (Fe) levels as well as bone mineral density (BMD) in the rats femur in the group 0.5% were similar to those in the group 0% (*P* > 0.05). At 1 month and 2 months after pseudopurpurin feeding, calcium (Ca), magnesium (Mg), zinc (Zn), and manganese (Mn) levels as well as bone mineral density (BMD) in the rats femur in the group 0.5% were significantly increased compared with those in the group 0% (*P* < 0.05). Iron (Fe) levels in the femur were not significantly different among the groups (*P* > 0.05) (Figure 2).

### 2.4. Histopathology of Internal Organs

**Figure 3:**
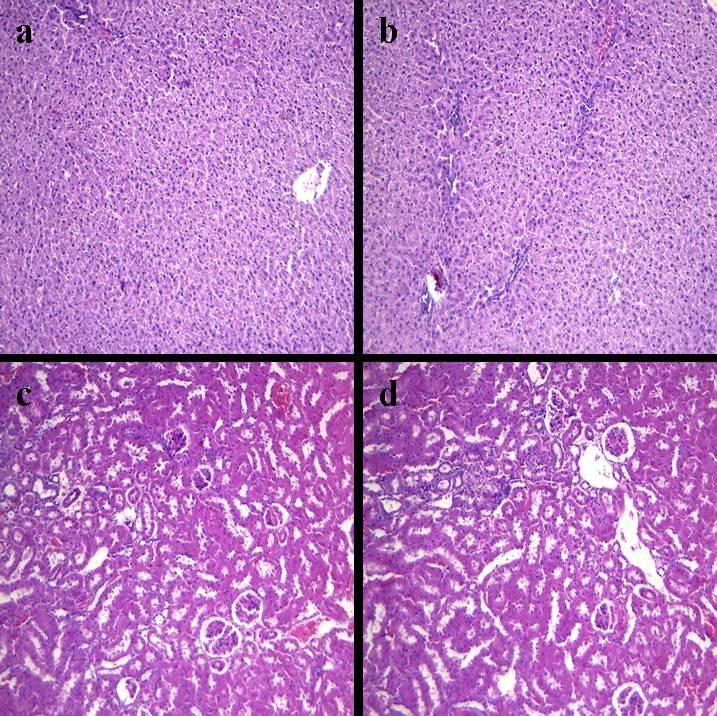
Liver sections of the group 0.5% (**a**) and the group 0% (**b**) and kidney sections of the group 0.5% (**c**) and the group 0% (**d**) are shown (hematoxylin and eosin (stain), 100×).

In the present study, no pathological changes were observed in the various organs of rats including heart, liver, spleen, lung, kidney, pancreas, stomach and small intestine in the groups 0% and 0.5% at 2 months after pseudopurpurin feeding (result of kidney and liver are shown in Figure 3, other data were not included).

### 2.5. Biochemical Evaluations

Biochemical evaluations showed the levels of liver duty enzyme and kidney duty enzyme in the blood plasma of rats in the 0% and 0.5% groups at 2 months after pseudopurpurin feeding. The levels of AST, ALT, BUN and CRE in blood plasma showed no significant differences among the 0% and 0.5% groups at 2 months after pseudopurpurin feeding (Table 2).

### 2.6. Femur Micro-CT

**Figure 4:**
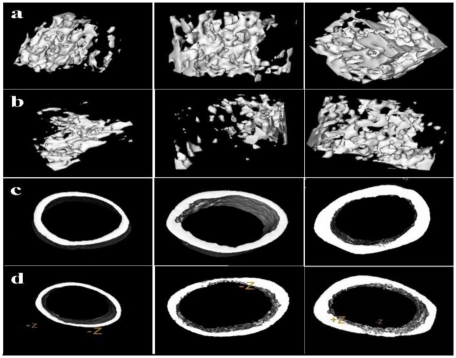
(**a**) Three-dimensional images of the distal femur in rats in the 0.5% group at 0.5, 1 and 2 months after pseudopurpurin feeding. (**b**) Three-dimensional images of the distal femur in rats in the 0% group at 0.5, 1 and 2 months after pseudopurpurin feeding. (**c**) Three-dimensional images of the femur diaphysis in rats in the 0.5% group at 0.5, 1 and 2 months after pseudopurpurin feeding. (**d**) Three-dimensional images of the femur diaphysis in rats in the 0% group at 0.5, 1 and 2 months after pseudopurpurin feeding.

The 3-D reconstruction of the bone structures and the quantification of trabecular and cortical bone changes in the femur by micro-CT are shown in Figure 4.

The mean thickness, inner perimeter, outer perimeter, marrow area, cortical area and total area of the femur diaphysis, together with the bone volume fraction (BV/TV), trabecular thickness (Tb.Th), trabecular number (Tb.N), trabecular separation (Tb.Sp) and structure model index (SMI) of the distal femur in the 0.5% groups were not significantly different compared to the 0% group at 0.5 months after pseudopurpurin feeding (all *P >* 0.05).

One month after pseudopurpurin feeding, BV/TV, Tb.Th and Tb.N were significantly increased and Tb.Sp and SMI were significantly decreased in the distal femur in the 0.5% groups compared with those in the 0% group (all *P <* 0.05). There were no differences in any of the parameters of the femur diaphysis among rats in the 0% and 0.5% groups (all *P >* 0.05).

Two months after pseudopurpurin feeding, the mean thickness, outer perimeter, cortical area, total area, inner perimeter and marrow area in the femur diaphysis, as well as BV/TV, Tb.Th and Tb.N in the distal femur in the 0.5% groups were significantly increased and Tb.Sp and SMI were significantly decreased compared with the 0% group (all *P <* 0.05) (Tables 3 and 4).

### 2.7. Affinity of Pseudopurpurin and Calcium

**Figure 5:**
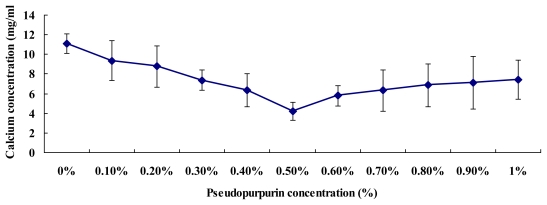
Each test tube showing residual calcium contents depending on the different dose of pseudopurpurin *in vitro*. Data are expressed as the means ± SD.

The chemical reaction of pseudopurpurin and calcium was complete, in each test tube the remaining calcium content was measured by chemical analyzers. Figure 5 shows the remaining calcium contents were lowest at a concentration of 0.5% pseudopurpurin, and then the remaining calcium contents began to rise with the increase of pseudopurpurin concentration *in vitro*. This demonstrated that the affinity of 0.5% pseudopurpurin and calcium ion is at its strongest compared with other concentrations of pseudo-purpurin *in vitro*.

### 2.8. Discussion

It has been known for many years that the bones of animals are colored red by feeding on the roots of the *Rubia tinctorum* L., which contains a number of coloring agents. However, Richter found that madder contained considerable quantities of pseudopurpurin, and considered that pseudopurpurin was mainly responsible for the vital staining of the bones of animals fed on madder, and was completely non-toxic to animals [14]. In the present study we showed that the data for calcium affinity depends on the dose of pseudopurpurin, and these results further demonstrated that at 0.5% pseudopurpurin the affinity of calcium ion is at its strongest compared to other concentrations of pseudopurpurin *in vitro*. Therefore, the rats were fed a new mixed pelleted diet in which 0.5% pseudopurpurin powder was added. Gross and microscopic examination of the kidney and liver showed that there was no pathological alteration in the rats in the 0.5% group compared to those in the 0% group. Concurrently, the levels of biochemical indicator in the blood plasma of rats in the 0.5% group were similar to the 0% group at 2 months after pseudopurpurin feeding. These results suggested that the administration of pseudopurpurin in the 0.5% group did not have significant toxic effects.

Our study is the first to demonstrate the beneficial effects of pseudopurpurin extract against reduction of bone mass and bone geometry architecture during growth and development of bone in rats. Bone is a very dynamic tissue which is constantly being repaired and renewed throughout life by the process of bone remodeling. The organic component of bone forms a framework upon which mineralization occurs. Bone mineral is composed mainly of calcium and phosphate, laid down in the form of hydroxyapatite [Ca_10_(PO_4_)_6_(OH_2_)] crystals [16]. In this study, Ca, Mg and Zn levels in the rats femur in the group 0.5% were significantly increased compared to those in the group 0% at 1 and 2 months after pseudopurpurin feeding. A rather complicated structural formula of the calcium-aluminum-alizarin compound was suggested by Rutishauser in 1940 [17]. Kiel and Heertjes [18–21] investigated the composition and structure of compounds formed by alizarin and its 3-derivatives with calcium, aluminum and various other metals. Pseudopurpurin resembles alizarin because it forms a colored metal salt that is highly insoluble in water [14]. Therefore, it is worth considering that the increasing deposition of principal salts in red bone is possibly associated with pseudopurpurin, which has a selective affinity for the principal salts of bone. Minerals formed *in vitro* have been found to consist of calcium and phosphorus deposited on well-banded collagen fibrils, with some of the crystals matured further into hydroxyapatite crystals [22,23]. Pseudopurpurin has a selective affinity for the principal salts of bone, and the pseudopurpurin-calcium salt, comes into contact with osteoid-tissue and the organic component of developing bone [14]. The pseudopurpurin-calcium salt has an adhesiveness which results in the calcium ion not being lost in bone metabolism. Calcium (Ca) is the major hydroxyapatite in bones [24]. Magnesium (Mg) is the second most abundant intracellular cation in vertebrates [25]. Magnesium can promote normal hydroxyapatite crystal growth [26]. In addition, trace elements are important in bone metabolism, as zinc and magnesium are closely bound to apatite crystals and zinc is essential for the alkaline phosphates in osteoblasts [27]. Zinc is bound to hydroxyapatite and mobilizes slowly during bone remodeling [28–31]. Therefore, pseudopurpurin increases bone mineral density (BMD) due to the high level of major mineral elements and trace elements present in the bone of rats [32].

BMD is the gold standard for evaluation of individuals at risk of osteoporosis, as it best predicts the fracture risk in people without previous fractures [33]. In the current study, the results manifested that BMD in the femur in the group 0.5% was significantly increased compared to that in the groups 0%. Measuring such microarchitectural parameters as the percent bone volume, trabecular bone structure, trabecular thickness, and trabecular separation may improve our ability to estimate bone strength [34–38]. In this study, the effects of a 0.5% dose of pseudopurpurin in the diet on femoral microarchitecture were investigated by scanning with micro-CT. The results of the micro-CT evaluation demonstrated that there were differences in all of the parameters of the femur among rats in the 0% and 0.5% groups at 2 months after pseudopurpurin feeding.

At one month after pseudopurpurin feeding, BV/TV, Tb.Th and Tb.N were significantly increased and Tb.Sp and SMI were significantly decreased in the distal femur in the 0.5% group compared with those in the 0% group. At two months after pseudopurpurin feeding, the mean thickness, outer perimeter, cortical area, total area, inner perimeter and marrow area in the femur diaphysis, as well as BV/TV, Tb.Th and Tb.N in the distal femur in the 0.5% group were significantly increased and Tb.Sp and SMI were significantly decreased compared with the 0% group. SMI of 0 and 3 represent bones that consist purely of plate- or rod-like structures, respectively [39]. The above results indicate that an increase of BMD and bone geometry architecture in the 0.5% group is related to the enhancement of the deposition of principal salts in red bone, which is possibly associated with a selective affinity of pseudopurpurin for the principal salts of bone. This suggests an adhesiveness of the calcium-dyestuff salts on the bone collagenous matrix so that not many calcium ions are lost during metabolism, promoting calcium salt mineralization deposition, and leading to enhanced structure and strength of bone. The overall quality of bone is determined by such characteristics as microarchitecture, geometry and material properties, all of which are affected by the rate of bone turnover [40].

## 3. Materials and Methods

### 3.1. Preparation of Pseudopurpurin

Commercially available madder powder was obtained from the Chinese Herbal Medicine Company (Kunming, Yunnan province, China). The dried madder root, dispersed in a mixture of 200 μL 1 mol·L^−1^ HCl solution and 200 μL methanol, was heated at 60 °C for 15 min with agitation. After addition of 10 mL water, the anthraquinones were extracted with 2 mL ethyl acetate. After a separation step and removal of the aqueous phase, evaporation of the solvent was performed under argon. The dry extract was then dissolved in 40 μL methanol before being injected on to the HPLC column.

### 3.2. Semi-Preparative HPLC

The impurities of pseudopurpurin were isolated using a Waters auto-purification system equipped with a 2525 binary gradient pump, a 2487 UV detector and a 2767 sample manager (Waters, Milford, MA, USA). A Water SunFire™ prep C 18 OBD™ column (150 mm × 30 mm i.d., particle size 10 μm) was used for semi-preparative isolation. Gradient elution was performed using a 20 mmol·L^−1^ ammonium acetate solution in water as mobile phase A and methanol as mobile phase B. The gradient composition was set from 0 to 95% B over 25 min and then to 100% B over 5 min; the 100% value was held for 20 min. The flow rate was set to 1 mL·min^−1^ and the injection volume was 1 mL. The column temperature was 30 °C and the detection wavelength was 255 nm. The sample solutions (~100 mg/mL) were prepared in the mobile phase.

### 3.3. Electrospray Ionization-Mass Spectrometry (ESI-MS)

An LTQXL system (Thermo, USA) with ESI ion source in the negative ion mode was used for qualitative analysis of the compounds. The separation was performed on an ACUITY UPLC^®^ BEH C18 column (1.7 μm, 50 mm × 3 mm i.d., Waters, USA). The linear gradient conditions were the same as those used for UPLC-UV analysis. Elution was performed at a solvent flow rate of 0.4 mL/min, and a portion of the column effluent (0.2 mL/min) was delivered into the ion source of the mass spectrometer. The conditions of MS analysis were as follows: Sheat Gas Flow Rate: 40, AUX Gas Flow Rate: 5, Sweep Gas Flow Rate: 1, Spray voltage (kv): 4; Capillary Temp (°C): 250, Capillary Voltage (V): 22.0, Tube Lens: 100.

### 3.4. Animals

All 1-month old female Wistar rats were acquired from the Experimental Animal Center in the First Clinical Hospital of Jilin University. All rats were fed in high efficiency particulate air (HEPA)-filtered isolation units at a constant 25 °C. Local government and the Medical Science Animal Experiment Committee of Jilin University agreed to use these rats for experiments. All rats in the the group 0% (*n* = 20), and the group 0.5% (*n* = 20) were used for this study, and were fed pelleted diets which 0% pseudopurpurin powder (group 0%) and 0.5% pseudopurpurin powder (group 0.5%) was added, and the group 0% was used as a control. Two group rats were fed a commercially available pelleted diet as prescribed by the manufacturer to fulfill all dietary needs. Rats in each group were sacrificed in batches at 0.5, 1 and 2 months after pseudopurpurin feeding by an overdose of sodium pentobarbital intraperitoneally and then their left femur were removed. At necropsy, the whole left femurs were first used for Dual energy X-ray absorptiometry (DEXA). The left femur diaphysis and the distal femur (*n* = 10, group 0.5%; *n* = 10, group 0%) were used prior to submission for micro-CT analysis. The left femurs (*n* = 10, group 0.5%; *n* = 10, group 0%) were used for an inductively coupled plasma optical emission spectrometer. Body weight and femur length were recorded.

### 3.5. Bone Mineral Elements

In all groups, the left femur samples from each rat were first cut into pieces with a ceramic knife to avoid metal contamination, and then the pieces obtained were dried under vacuum at 50 °C to remove water and some volatile organic components. Samples after pretreatment were weighed (0.5 g) precisely into 30 mL vessels and 5 mL of high purity HNO_3_ was added. The vessels were then placed on a heating furnace with the temperature set at 120 °C until clean solutions were obtained. After cooling, the solutions were transferred into 25 mL flasks and fixed to volume with ultrapure water. An inductively coupled plasma optical emission spectrometer (ICP-OES, iCAP6300, ThermoScientific, USA) was employed for analysis of major elements.

### 3.6. Dual Energy X-ray Absorptiometry

All left femurs were assessed for Dual energy X-ray Absorptiometry (DEXA). Prior to testing, they were slowly brought to room temperature in a saline bath. Scanning was performed with each bone positioned on its caudal surface, and bone mineral density (BMD; mg/cc) was collected from femur diaphysis of all rats.

### 3.7. Histopathological Analysis

Kidneys, livers, hearts, spleens, stomachs and lungs samples were harvested from rats in all groups at 2 months after treatment, and were fixed in 10% neutral buffered formalin and embedded in paraffin for histopathological analysis. The tissue samples were sectioned at 4 μm. Cross-sectioned tissues were stained with hematoxylin and eosin. The following tissues were examined by light microscopy for the presence of lesions.

### 3.8. Detection of Blood of Rats

The blood samples containing anticoagulant heparin sodium were used for the detection. The separated plasma from the remaining blood was used for the detection of the aspartate aminotransferase (AST), alanine aminotransferase (ALT), blood urea nitrogen (BUN), and creatinine (CRE) using the Beckman Synchron CX7 Delta Chemistry Analyzers (Beckman, USA). Each detection was performed in triplicate.

### 3.9. Micro-CT Scanning

After euthanasia, the left femurs of all rats were scanned by a desktop micro-CT system (eXplore Locus SP, GE Healthcare, USA). For image acquisition, the specimens were incised into cylinders 20 mm in diameter. The specimens were scanned with 14 μm isotropic voxel size using a large tube-14 μm-150 min-ss-micro-tomography scan protocol. The scan protocol consisted of rotation through 210° at a rotation step of 0.4°, X-ray settings were standardized to 80 kV and 80 μA, and the exposure time was 2960 ms per frame. The scan time was approximately 150 min per sample. Three-dimensional (3-D) surface renderings were performed using Mic-view V 2.1.2 3-D reconstruction software. Regions of interest (ROIs) were reconstructed and analyzed by micro-CT with the same thresholds. The database was analyzed, which led to 3-D parameters of each ROI. Each pair of these parameters in the same specimen was compared for each group.

### 3.10. Affinity of Pseudopurpurin and Calcium Test

The red calcium salt of pseudopurpurin floated on water and was formed in neutral solution. To investigate the affinity of pseudopurpurin and calcium ions, to each test tube was added 10 mL 10 mM CaCl_2_ (pH 7.0) and 1 mL different dose of pseudopurpurin. The doses of pseudopurpurin were 0, 0.1, 0.2, 0.3, 0.4, 0.5, 0.6, 0.7, 0.8, 0.9 and 1%, respectively. These samples were homogenized and centrifuged (5000 *g*) for 15 min at 4 °C. The upper solid phase of the calcium salt of pseudopurpurin was removed, and the lower aqueous phase with remains of the calcium ions was measured by the Beckman Synchron CX7 Delta Chemistry Analyzers (Beckman, USA). Each detection was performed in triplicate.

### 3.11. Statistical Analyses

All data are expressed as mean values ± S.D. Data were considered statistically significant at *P <* 0.05 using paired *t*-test. All statistical analyses were performed using the statistical package SPSS for Windows version 11.

## 4. Conclusions

According to the above results, we consider that adding pseudopurpurin to the diet of rats at a concentration of 0.5% is most suitable. The feeding of 0.5% pseudopurpurin which has specific chemical affinities for calcium for bone improvement and level of bone mineral density enhances the geometry architecture compared with the 0% group.

## Figures and Tables

**Table 1:** Body weight and femur length of rats at 0.5, 1 and 2 months after pseudopurpurin feeding.

		Body weight (g)	Femur length (cm)
0.5% group	0.5 month	24.5 ± 5.66	0.81 ± 0.057
	1 month	57.3 ± 9.37	1.78 ± 0.72
	2 months	123.6 ± 22.2	2.12 ± 0.92
0% group	0.5 month	23.8 ± 6.01	0.79 ± 0.034
	1 month	54.6 ± 11.57	1.55 ± 0.83
	2 months	116 ± 21.5	1.94 ± 0.94

**Table 2:** Comparison of the biochemical levels in blood plasma of rats in 0.5% and 0% groups at 2 months after pseudopurpurin feeding.

	AST (U/L)	ALT (U/L)	BUN (mmol/L)	CRE (umol/L)
0.5% group	82.40 ± 5.47	31.24 ± 3.17	11.52 ± 1.44	22.38 ± 1.44
0% group	83.24 ± 5.99	31.45 ± 2.66	10.79 ± 1.24	21.02 ± 1.54

Data are expressed as the means ± SD;

**Table 3:** 3-D micro-structural properties of the distal femur in rats in groups 0.5% and 0%.

		BV/TV (%)	Tb.Th (mm)	Tb.N (/mm)	Tb.Sp (um)	SMI
0% group rats	0.5 month	2.32 ± 0. 3	0.051 ± 0.009	2.76 ± 0.58	0.365 ± 0.03	2.22 ± 0.3
0.5% group rats		2.38 ± 0.5	0.054 ± 0.01	2.77 ± 0.69	0.370 ± 0.037	2.20 ± 0.4
0% group rats	1 month	3.16 ± 0.04	0.08 ± 0.007	3.16 ± 1.11	0.295 ± 0.013	1.92 ± 0.03
0.5% group rats		3.82 ± 0.03 *	0.13 ± 0.007 *	4.01 ± 1.07 *	0.216 ± 0.014 *	1.30 ± 0.02 *
0% group rats	2 months	5.61 ± 0.08	0.15 ± 0.05	3.99 ± 1.0	0.218 ± 0.003	1.41 ± 0.011
0.5% group rats		6.75 ± 0.08 *	0.23 ± 0.04 *	5.16 ± 1.3 *	0.167 ± 0.005 *	0.86 ± 0.01 *

Data are expressed as the means ± SD;

* *P* < 0.05 *versus* 0% group rats.

Bone volume fraction (BV/TV), trabecular thickness (Tb.Th), trabecular number (Tb.N), trabecular separation (Tb.Sp) and structure model index (SMI).

**Table 4:** 3-D micro-structural properties of the mid-femur diaphysis in rats in groups 0.5% and 0%.

		Mean Thickness (mm)	Inner Perimeter (mm)	Outer Perimeter (mm)	Marrow Area (mm^2^)	Cortical Area (mm^2^)	Total Area (mm^2^)
0% group rats	0.5 month	0.17 ± 0.03	4.21 ± 0.9	5.7 ± 1.1	2.76 ± 0.08	0.71 ± 0.03	3.22 ± 0.7
0.5% group rats		0.18 ± 0.05	4.42 ± 1.0	6.34 ± 1.1	2.77 ± 0.09	0.718 ± 0.037	3.20 ± 0.8
0% group rats	1 month	0.241 ± 0.05	6.57 ± 1.0	8.28 ± 1.54	3.34 ± 0.67	1.86 ± 0.36	5.33 ± 1.64
0.5% group rats		0.267 ± 0.03	6.82 ± 0.88	8.41 ± 1.33	3.54 ± 0.78	1.99 ± 0.34	5.35 ± 1.55
0% group rats	2 months	0.38 ± 0.09	7.56 ± 1.82	10.87 ± 3.1	4.01 ± 1.64	3.75 ± 1.22	7.16 ± 2.31
0.5% group rats		0.43 ± 0.07 *	8.41 ± 1.34 *	13.88 ± 2.87 *	5.17 ± 1.77 *	4.8 ± 1.12 *	8.13 ± 2.13 *

Data are expressed as the means ± SD;

* *P* < 0.05 *versus* 0% group rats.
